# Development and Stability Evaluation of Oleogel-in-Water Emulsions Using Whey Protein Isolate–Ferulic Acid Nanoparticles for Ganoderma Lucidum Spore Oil Encapsulation

**DOI:** 10.3390/gels12080735

**Published:** 2026-08-17

**Authors:** Wenjia Yan, Yuting Bao, Hao Wang, Shanshan Xu, Chengfang He, Zhuochen Wang, Jian Jiang

**Affiliations:** 1 Institute of Agro-Products Processing, Anhui Academy of Agricultural Sciences, Hefei 230001, China; 13681126407@163.com (W.Y.); whdamonxin@163.com (H.W.); xss8894@163.com (S.X.); hecf1616@163.com (C.H.); 2 Anhui Engineering Laboratory of Food Microbial Fermentation and Functional Application, Hefei 230001, China; tina_y_ting@163.com

**Keywords:** whey protein isolate, oleogel, emulsion, glossy ganoderma spore oil

## Abstract

This work evaluated how ferulic acid (FA) impacted the conformational properties of whey protein isolate (WPI) and altered the environmental tolerance of oleogel/water (Og/W) emulsions formulated with *Ganoderma lucidum* spore oil (GLSO). Molecular dynamics simulation analyses revealed that FA interacted with α-lactalbumin via hydrogen bonding and hydrophobic interactions, whereas it bound into the hydrophobic cavity of β-lactoglobulin through a “lock-and-key” mode driven primarily by hydrophobic forces. Fourier transform infrared spectroscopy analysis verified that such non-covalent forces triggered the dissociation and structural extension of WPI, which was manifested as a significant loss of α-helix and β-sheet architectures along with a corresponding rise in random coils. FA addition increased the positive charge, mean droplet size, interfacial contact angle and antioxidant stability of WPI-FA nanoparticles when the WPI-to-FA ratio exceeded 1:2 (i.e., WPI was in excess relative to FA). The GLSO-based Og/W emulsions exhibited a weak gel structure with predominantly elastic characteristics. Furthermore, WPI-FA nanoparticles fabricated at a 2:1 ratio minimized emulsion droplet size and imparted optimal stability to the Og/W emulsions, demonstrating superior freeze–thaw and salt resistance, alongside suppressed GLSO flavor release. This work provides critical insights into tailoring protein-polyphenol interactions to stabilize GLSO-based Og/W emulsion delivery systems for food applications.

## 1. Introduction

*Ganoderma lucidum* spore oil (GLSO) is a lipid-rich extract obtained from sporoderm-broken *G. lucidum* spores [1]. It comprises a complex array of bioactive constituents, primarily triterpenoids, sterols, and unsaturated fatty acids (UFAs) [2]. Recent studies have demonstrated that GLSO possesses diverse bioactivities, including immunomodulatory, anti-inflammatory, neuroprotective, and hypolipidemic effects, as well as potential benefits in hepatoprotection and leukemia chemoprevention [3,4,5]. Despite its therapeutic potential, GLSO is highly susceptible to oxidative degradation. Exposure to light, elevated temperatures, and oxygen triggers the rapid oxidation of UFAs and triterpenoids, leading to the formation of off-flavors and a significant reduction in bioactivity [6,7]. Furthermore, the hydrophobic nature of GLSO results in poor aqueous solubility and dispersibility, which ultimately limits its gastrointestinal absorption and systemic bioavailability [8]. Consequently, developing effective strategies to enhance the oxidative stability and oral delivery of GLSO is essential for its broader application in the functional food and nutraceutical industries. Given the challenges in retaining and utilizing bioactive molecules in specialty oils, the employment of oil-in-water emulsions has been extensively recommended [9,10]. Owing to their structural resilience and protective capabilities, these emulsion-based platforms are exceptionally qualified for the ambient encapsulation, transport, and storage of GLSO, thereby affording elevated chemical protection and gastrointestinal accessibility [11].

Conventional oil-in-water (O/W) emulsions, including nano-emulsions, coarse emulsions, emulsion gels and high internal phase emulsion, have been extensively utilized across the food, pharmaceutical, and cosmetic industries [12,13,14,15]. In recent years, oleogel-in-water (Og/W) emulsions have emerged as a novel emulsion architecture, wherein the dispersed oil phase is an oleogel and the continuous phase is an emulsifier solution [16,17]. Oleogels are structured oils in which a gelator forms a three-dimensional network, transforming liquid oil into a solid-like form [18]. This structural modification provides a robust platform for encapsulating and protecting functional oils against harsh environmental conditions. Previous studies have demonstrated that Og/W emulsions possess a superior capacity to preserve the activity of functional oils as compared with traditional emulsions. Wang et al. (2024) [19] reported that incorporation of 3% hemp seed protein bolstered the resistance of Og/W emulsions against environmental challeges (e.g., thermal treatment, ionic stress, and pH shifts) over conventional counterparts. The camellia oil-based Og/W systems fabricated by Pan and co-workers (2021) [20] via monoglyceride laurate gelation displayed exceptional oxidative and thermal stability. Even so, comprehensive knowledge regarding the processing and physicochemical properties of GLSO-based Og/W emulsions is still lacking and requires targeted research.

Previous research on Og/W emulsions primarily focuses on how oleogel-phase composition (e.g., gelator type and concentration) impacts the physical and chemical properties of emulsions, while the emulsifier component remains largely restricted to single-protein system [21,22,23]. The non-covalent assembly of protein-phenolic complexes has attracted considerable scientific interest of late, as it not only enhances the intrinsic properties of the proteins but also broadens the functional layout of these co-ingredients in food matrices [24]. Prior research indicates that soybean protein non-covalently binds with proanthocyanidins via hydrophobic interactions and hydrogen bonds. The resulting nanocomplexes effectively stabilize O/W Pickering emulsions, leading to enhanced emulsion stability and controlled-release performance [25]. The interaction between polyphenols and proteins fundamentally dictates interfacial behavior and overall emulsion functionality [26,27,28]. Therefore, clarifying protein–polyphenol interactions and their regulatory roles in Og/W emulsions structured with GLSO is essential for the rational design of stable and high-value functional foods. Nevertheless, the application of non-covalently bound protein-polyphenol complexes to stabilize Og/W emulsions remains an under-explored territory in food science.

Whey protein isolate (WPI) is a high-performance emulsifier with balanced hydrophilic-lipophilic properties. It can stabilize emulsions by adsorbing onto the oil-water interface of Og/W emulsion and forming a cohesive and rearranged interfacial film [29]. β-Lactoglobulin (β-LG) and α-lactalbumin (α-LA) constitute the bulk of WPI, holding individual shares of 65% and 25%. Ferulic acid is a major phenolic acid component of cereal grains. It exerts several beneficial biological effects, including the ability to reduce blood sugar, lower hypertension, and mitigate inflammation [30]. Extensive evidence supports the modulatory effects of FA on food proteins. Specifically, Chen et al. (2024) [31] revealed that FA triggers soybean protein unfolding and depolymerization through hydrogen bonds and hydrophobic effects, concurrently increasing random coil and β-turn contents. Xue et al. (2023) [32] confirmed that conjugating FA with β-lactoglobulin (β-LG) enhances emulsification and concurrently reduces β-LG allergenicity. Taken together, these findings suggest that nanoparticles fabricated via WPI–FA non-covalent conjugation could be favorably adopted for constructing GLSO-based O/W emulsions, with a potential upside in stability. Nonetheless, in-depth research on this front is still lacking.

This study focused on the concentration-dependent impacts of FA on the molecular structures and functionalities of WPI within the formed WPI-FA non-covalent nanoparticles. WPI-FA nanoparticle was used as the emulsifier to stabilize GLSO-based Og/W emulsion. The microstructure, stability, and intelligent sensory analysis properties of GLSO-based Og/W emulsion was systematically evaluated. The findings provide a theoretical foundation for high-performance functional lipid delivery systems. Furthermore, this work guides the strategic application of polyphenols in food matrices.

## 2. Results and Discussion

### 2.1. Turbidity, Mean Particle Size, ζ-Particle and Wettability of WPI-FA Nanoparticles

**Figure 1:**
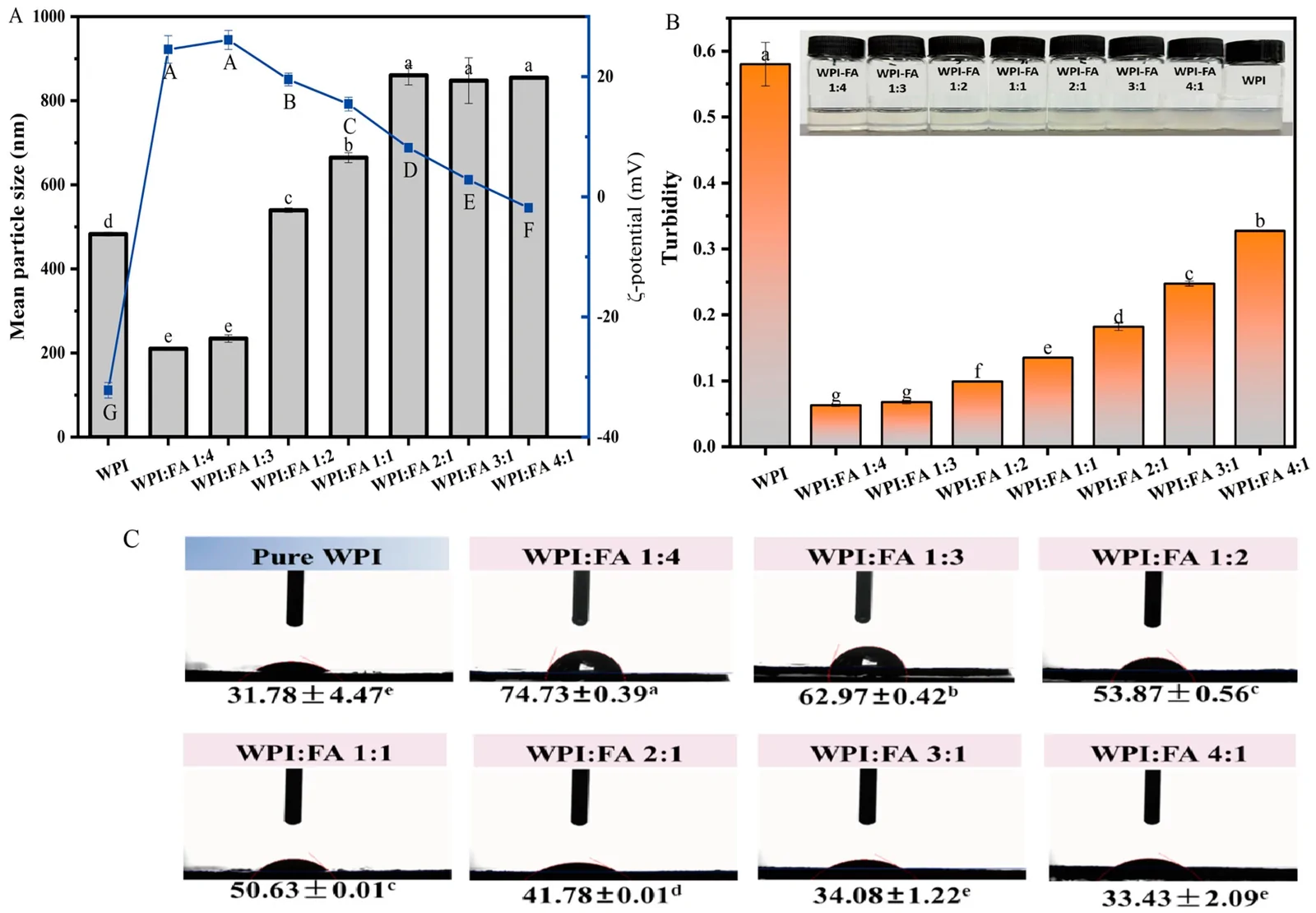
The physicochemical properties of WPI-FA nanoparticles. (**A**) Mean particle size and ζ-potential; (**B**) turbidity; (**C**) wettability. Means of the same parameter with different letters are significantly different (n = 3, *p* < 0.05).

The particle size of WPI-FA nanoparticle was larger than that of WPI particles when the FA content was below a WPI:FA ratio of 1:2. Nevertheless it became smaller than that of WPI particles at or above this ratio. Particle-size measurements revealed a decreasing trend with increasing FA content (Figure 1A), which was consistent with the potential reported by Thongkaew et al. (2014) [33]. The reduction in particle size weakened light scattering, thereby markedly decreasing the visual and analytical turbidity of the WPI-FA nanoparticle (Figure 1B). The ζ-potentials of WPI were negative owing to the negative charge on the surfaces of the WPI molecules above the isoelectric point. Furthermore, at a WPI/FA mass ratio of approximately 4:1, the ζ-potential of the nanoparticles was close to zero (Figure 1B). The near-electroneutral state may attenuate electrostatic repulsion between particles, rendering them more prone to aggregate and thereby increasing the overall particle size [34]. This result was consistent with previous study [35], that high aggregation of soybean protein molecules occurred under the cross-linking of polyphenol. Moreover, FA addition elevated the positive charge and the absolute ζ-potential of the samples. The resulting increase in electrostatic repulsion enhanced system stability, keeping the WPI-FA nanoparticles finely dispersed with a small particle size. The oil–water interfacial contact-angle of the nanoparticles increased significantly with rising FA content, indicating an enhancement in system hydrophobicity [36] (Figure 1C). This shift implied that the incorporation of FA may strengthen the interfacial activity of WPI-based nanoparticles, thereby improve their emulsification performance when apply as emulsifiers.

### 2.2. FT-IR Analysis and Secondary Structure of WPI-FA Nanoparticles

**Figure 2:**
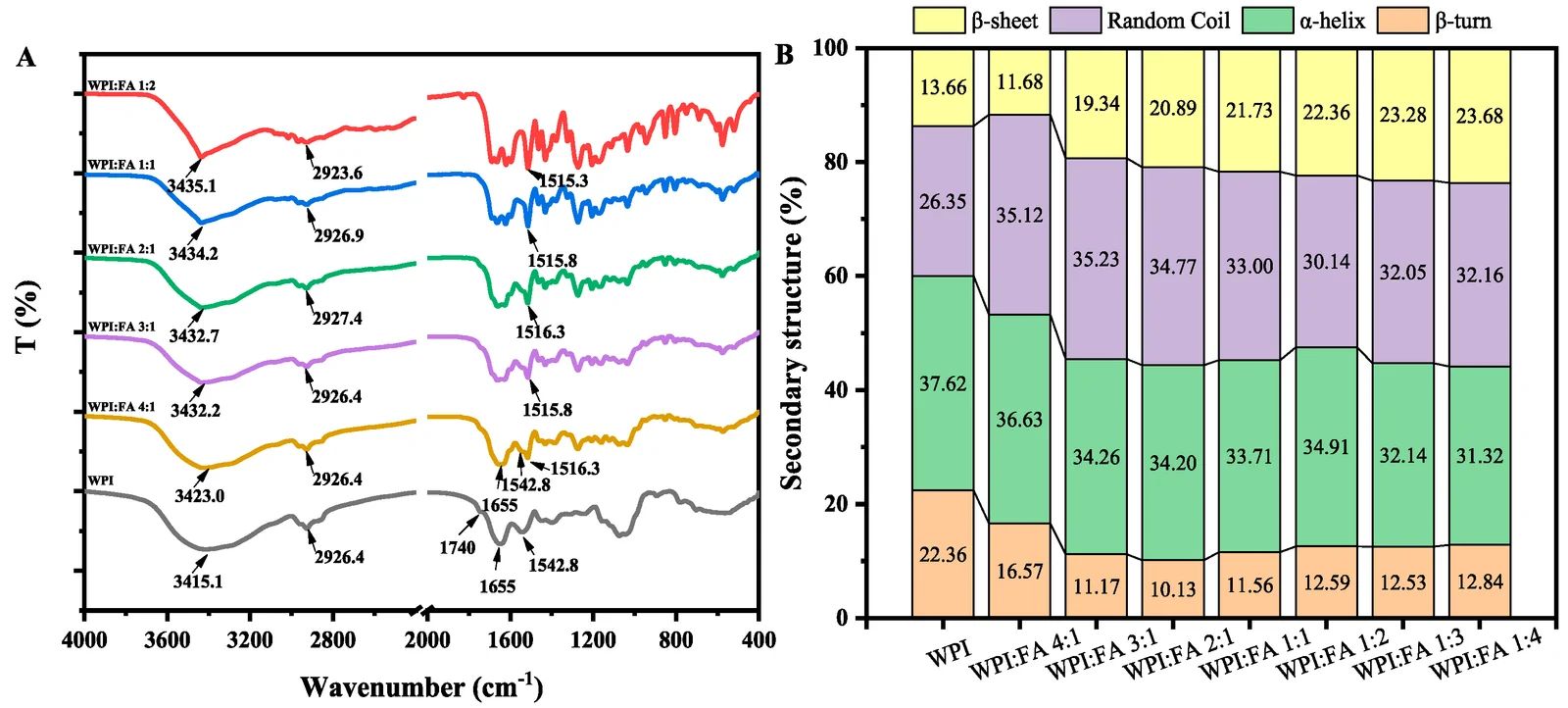
FTIR spectroscopic analysis of WPI and WPI-FA nanoparticles. (**A**) FTIR spectra; (**B**) secondary structure composition of nanoparticles.

A blue shift was observed for the O–H stretching vibration at 3400 cm^−1^, indicating that enhanced hydrogen-bonding interactions between WPI and FA within the system. With increasing FA proportion, the band at 2926 cm^−1^ attributed to alkyl (C-H) stretching exhibited a blue shift, suggesting that protein conformational rearrangements occurred during WPI–FA interactions. Within the amide I region (1600~1700 cm^−1^), the stretching vibrations of the backbone C=O bonds serve as key indicators of conformational variations. Specifically, the sub-bands centered at 1610~1640 cm^−1^, 1640~1650 cm^−1^, 1650~1660 cm^−1^, and 1660~1695 cm^−1^ correspond to β-sheet, random coil, α-helix, and β-turn conformations, respectively [37]. The quantified proportions of these secondary motifs for both native WPI and WPI–FA nanoparticles are summarized in Figure 2B. Evidently, the introduction of FA altered WPI to depolymerization and unfolding, evidenced by a distinct reduction in β-sheet and α-helix fractions alongside a concomitant increase in random coil contents. This trend aligns with the observations of Hasni et al. (2011) [38], who demonstrated that the conjugation of tea polyphenols to β-lactoglobulin diminished the α-helix and β-sheet components while elevating the disordered structures. According to Chen et al. (2024) [31], FA induced the breakdown and denaturation of soybean protein isolate, with hydrogen bonding and hydrophobic interactions serving as the main driving forces. As a result, the levels of disordered structures of protein such as random coils are significantly enhanced.

### 2.3. SEM Micrograph of Different WPI-FA Nanoparticles

**Figure 3:**
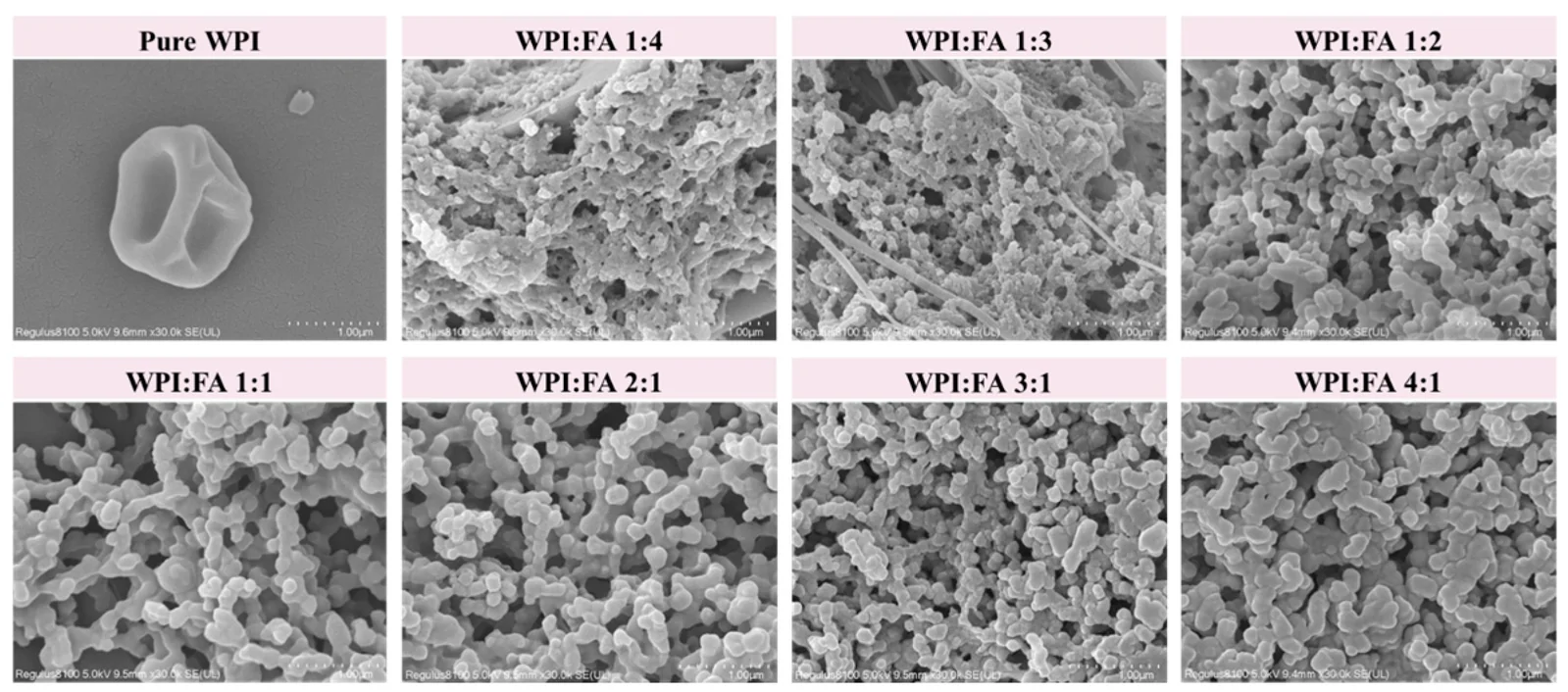
SEM micrograph of different WPI-FA nanoparticles.

SEM observations at diverse magnifications revealed the surface microstructures of the samples (Figure 3). The WPI exhibited a largely near-spherical granular microstructure, in agreement with previous reports [39]. The size of WPI-FA nanoparticles exhibited a decreasing trend with increasing FA concentration. A pronounced particle aggregation tendency was observed, which implied possible conformational changes in the protein. Yang et al. (2020) [40] have reported a similar phenomenon, which they ascribed to the hydrophobic interaction between β glucan and polyphenols. This interaction promoted protein clustering and thus generated a three-dimensional skeletal structure.

### 2.4. Molecular Docking

**Figure 4:**
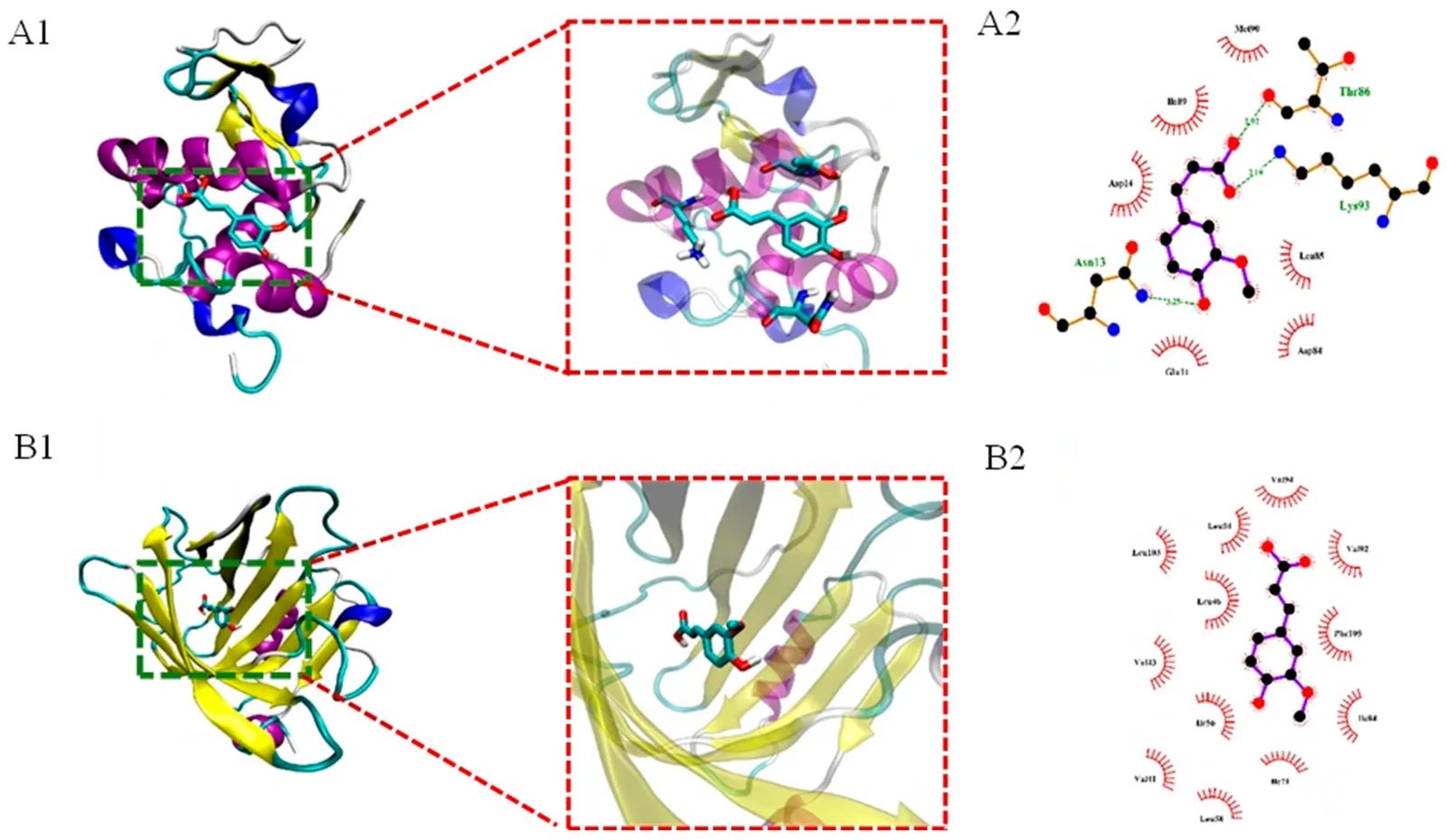
Molecular docking analysis of interactions included: (**A1**) the binding sites for FA on α-La; (**B1**) the binding sites for FA on β-LG; (**A2**) 2D diagrams of the week interaction between FA and α-La; (**B2**) 2D diagrams of the week interaction between FA and β-LG.

Molecular docking simulations serve as a visualization approach for elucidating the binding forces, interaction sites, and associated energies in protein–small molecule interactions [41]. Molecular docking analysis was performed to gain insights into the binding mechanisms of FA toward the two major proteins in WPI, namely α-lactalbumin (α-LA) and β-lactoglobulin (β-LG). The minimum binding energy was −5.05 ± 0.07 kcal/mol and −6.40 ± 0.01 kcal/mol, respectively, indicating a better affinity between FA and β-LG. As illustrated in Figure 4A, the α-LA formed strong hydrophobic interactions with the residues Met-90, Ile-89, Asp-14, Leu-85, Asp-84 and Glu-11 through the corresponding carbon atom of the FA molecule. Thr-86 (2.92 Å), Asn-13 (3.25 Å) and Lys-93 (3.19 Å) of α-LA formed hydrogen bonds with FA. The results indicated that the binding between α-LA and FA was primarily driven by hydrogen bonding and hydrophobic interactions. The FA molecule was found to be deeply embedded in the preferred binding pocket of β-LG, lying near several reactive residues (Figure 4B). The hydrophobic forces dominated in FA/β-LG nanoparticles. The hydrophobic interactions were formed between FA and Val-94, Val-92, Phe-105, Ile-84, Ile-71, Ile-56, Leu-46, Leu-54, Leu-103, Val-43, Val-41 and Leu-58 residues in β-LG.

### 2.5. Molecular Dynamics Simulation

**Figure 5:**
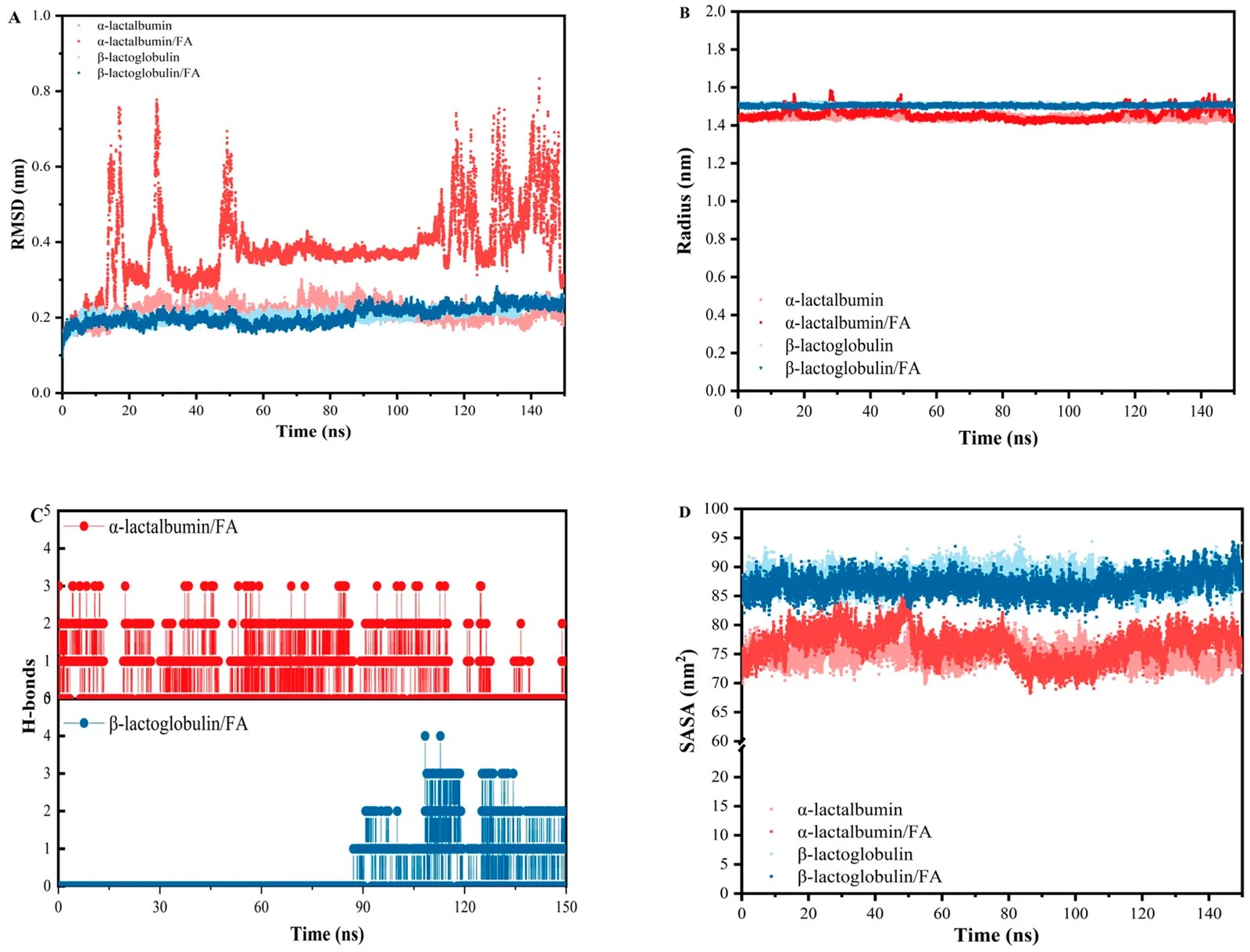
Radius (**A**), root mean square deviation (RMSD) (**B**), solvent accessible surface area (SASA) (**C**) and number of hydrogen bonds (**D**) of interaction of whey proteins α-LA/β-LG with ferulic acid during molecular dynamics simulations.

Monitoring the root-mean-square deviation (RMSD) over time is essential for assessing structural stability. Lower RMSD values indicate more stable conformations. Over 150 ns, both β-LG and the β-LG/FA complex exhibited RMSD fluctuations within 0.16–0.27 nm (Figure 5A). Between 15 and 80 ns, the β-LG/FA complex showed slightly lower RMSD than free β-LG, indicating that FA binding moderately enhances β-LG stability. From 80 to 90 ns, the slow rise in RMSD indicated that the protein undergone conformational adjustments as the FA ligand approached or initially entered the binding pocket. After 90 ns, the RMSD plateaued, implying a tight lock-and-key binding between FA and the protein with no subsequent conformational changed. In contrast, the α-LA/FA complex displayed significantly higher RMSD than the β-LG/FA complex during the 56–105 ns equilibration period, suggesting greater conformational rearrangements. After 105 ns, persistent RMSD fluctuations and eventual molecular dissociation confirmed that α-LA and FA did not form a stable complex, reflecting weak intermolecular interactions.

The radius of gyration (Rg), derived from the backbone atoms during the 150 ns trajectory, is presented in Figure 5B as a measure of structural compactness. Typically, a smaller Rg signifies a densely folded architecture, whereas larger values point to a more open, loosely organized conformational state. For α lactalbumin, the Rg remained stable around 1.45 nm after the initial 10 ns equilibration, demonstrating a highly compact and well-maintained globular fold. Upon FA binding, the Rg increased slightly to approximately 1.48 nm and exhibited more noticeable fluctuations. This suggested that FA binding induced a modest loosening of the α lactalbumin structure, although the protein remained essentially compact. The β Lg exhibited a consistently higher Rg of about 1.50 nm throughout the simulation, reflecting a natively more expanded and flexible conformation compared to α lactalbumin. No significant conformational change in β Lg was observed upon FA addition, given that both the mean Rg and the associated fluctuations remained largely unaltered. This observation was fully consistent with the molecular docking results, which showed that FA inserted directly into the preexisting hydrophobic cavity of β-Lg without inducing a substantial conformational rearrangement. The lack of Rg change therefore supported a “lock and key” type binding mode for β-Lg, in contrast to the slight expansion observed for α-lactalbumin upon ligand binding.

Hydrogen bonds are crucial for maintaining protein stability. Figure 5C shows the number of hydrogen bonds formed between individual molecules. For α-LA/FA composite, hydrogen bonds were formed throughout the entire simulation from 0 ns to 149.12 ns, but the number of hydrogen bonds exhibited pronounced fluctuations. In contrast, the β-Lg/FA composite only started forming hydrogen bonds between 90 ns and 150 ns, and once established, the hydrogen bond counts remained relatively stable with only minor fluctuations. In line with the results of RMSD and Rg, FA formed a network of hydrogen bonds with specific amino acid residues inside the β protein pocket, thereby immobilizing FA within the pocket.

The solvent-accessible surface area (SASA) quantifies the molecular surface exposed to the surrounding solvent. For α-lactalbumin, the free form maintained a constant SASA of 72.08 nm^2^, whereas the α-lactalbumin/FA complex remained constant at 78.00 nm^2^ (Figure 5D). This indicated that FA molecule induced increase in solvent exposure throughout the simulation. A marked reduction in SASA fluctuations was observed between 77 and 116 ns, suggesting an unstable complex for α-lactalbumin/FA complex. This finding was consistent with the corresponding RMSD, Rg and H bonds analyses. Free β-lactoglobulin exhibited a linear SASA increase from 83 to 88 nm^2^ during the first 3 ns, reflecting gradual unfolding before reaching a plateau. In contrast, the FA-bound protein maintained a nearly constant SASA of about 87 nm^2^. This indicates that FA binding does not substantially alter the absolute solvent accessibility, but instead locks β-Lg into a kinetically stable conformation.

### 2.6. Antioxidant Stability of WPI-FA Nanoparticles

The incorporation of FA into WPI significantly enhanced the antioxidant capacity of the resulting composite nanoparticles, as evaluated by DPPH and ABTS radical scavenging assays, as well as total antioxidant capacity (T-AOC). Both DPPH and ABTS scavenging activities increased with rising FA proportion and reached a plateau at a WPI/FA ratio of 1:2, indicating that this ratio represented an optimal balance for free radical inhibition. In contrast, T-AOC exhibited a continuous increase up to the highest tested ratio of 1:4, suggesting that the overall reducing capacity follows a different dose–response pattern. These results demonstrated that complexation with FA markedly improved the antioxidant properties of WPI in a generally dose-dependent manner, with assay-dependent variations in the saturation point. The pronounced radical scavenging capacity of the WPI-FA nanoparticle is presumably attributable to the potent antioxidant efficacy intrinsic to FA. This synergistic effect corroborated the earlier observations of Zhu et al. (2024) [42], who similarly documented enhanced antioxidant performance following the conjugation of FA with coconut protein.

### 2.7. Salt Ion Stability of WPI-FA Nanoparticle

**Figure 6:**
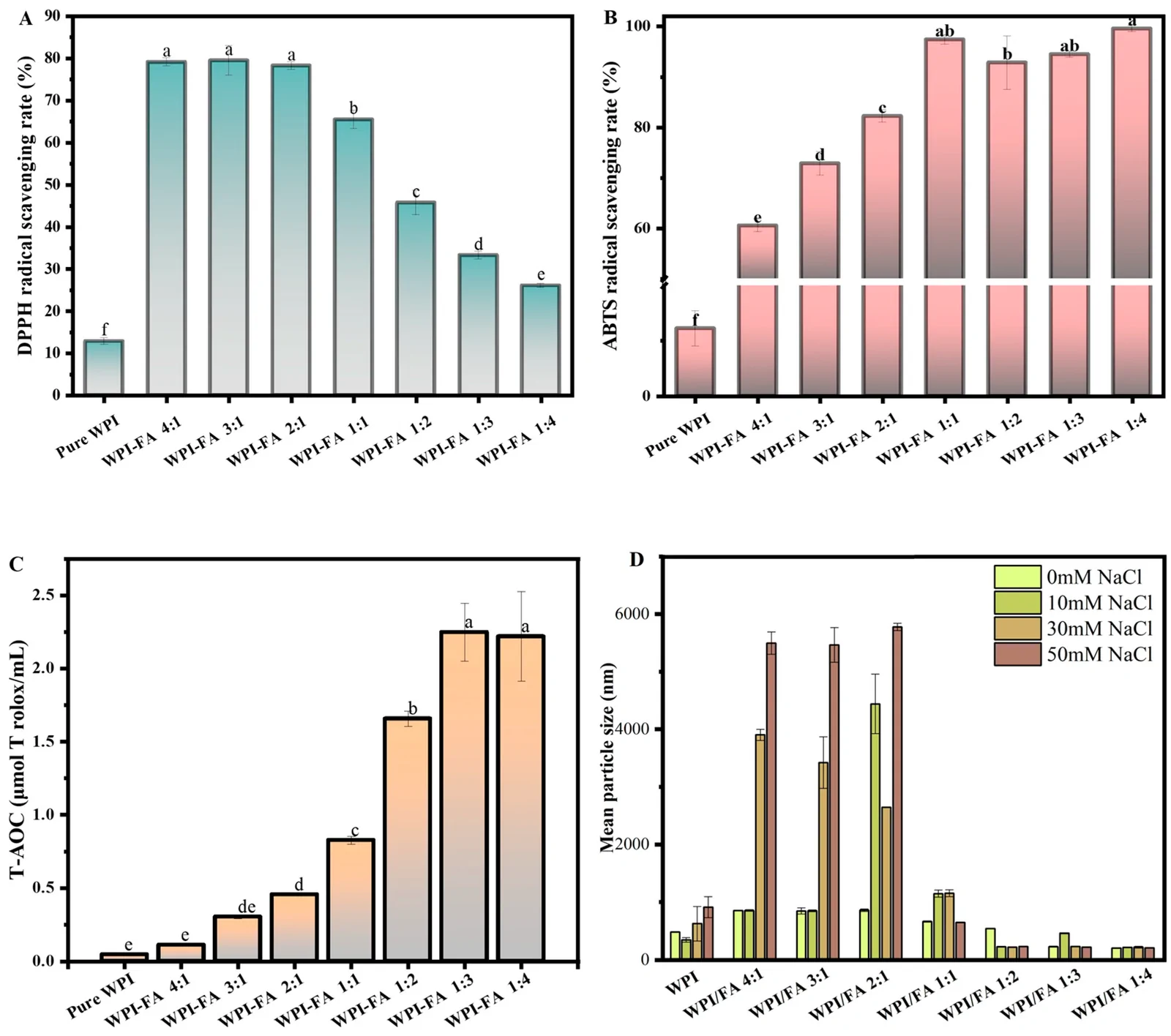
The antioxidant stability and salt ion stability of WPI-FA nanoparticles. (**A**) DPPH radical scavenging rate; (**B**) ABTS radical scavenging rate; (**C**) T-AOC; (**D**) Mean particle size of the nanoparticles at different salt concentrations. Different letters indicate *p* < 0.05 among samples.

As salt is a common constituent of food systems, assessing the stability of WPI-FA nanoparticles across different ionic strengths is necessary. The mean particle size of WPI-FA nanoparticles as a function of ionic strength (0–500 mM) is presented in Figure 6D. WPI nanoparticles size increased significantly with increasing salt ion concentration. For WPI/FA ratios of 2:1, 3:1, and 4:1, the particle size increased significantly with increasing salt ion concentration. The WPI/FA ratio of 1:1 exhibited a different trend. When the salt ion concentration increased to 50 mM, the particle size decreased instead. For the WPI/FA ratios of 1:2, 1:3, and 1:4, there is less changes in the particle size with increasing salt ion concentration. This indicated that these WPI-FA nanoparticles with rations of 1:2, 1:3, and 1:4 exhibited the higher salt stability.

### 2.8. The Freeze–Thaw Stability of the Og/W Emulsion Stabilized by WPI-FA Nanoparticle

**Figure 7:**
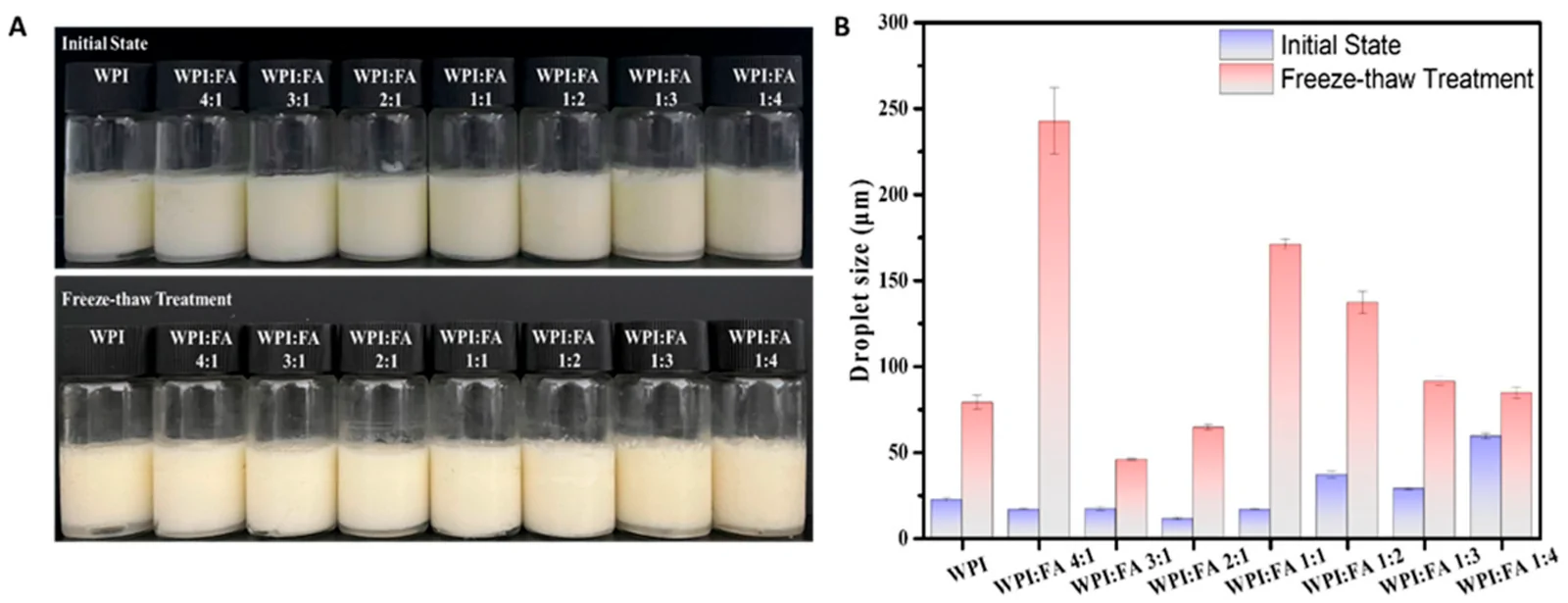
The freeze-thaw stability of Og/W emulsions stabilized by different nanoparticles. (**A**) The apparent diagrams; (**B**) average particle sizes of the emulsion before and after freeze-thaw treatment.

As illustrated in Figure 7A, the WPI and WPI–FA nanoparticles successfully stabilized the GLSO-based Og/W emulsions, which exhibited homogeneous, white, and opaque appearances. With increasing FA content, the average droplet size firstly decreased and then increased, reaching its minimum at a WPI/FA ratio of 2:1 (Figure 7B). After repeated freeze–thaw cycles, no macroscopic oil separation was observed at the top of any emulsion sample, despite a consistent increase in their particle sizes. During the gradual freezing process, crystallization within one droplet can cause it to penetrate the fluid domain of an adjacent droplet, thereby initiating the formation of irregular aggregates. Upon thawing, these partially crystallized droplets melt and undergo fusion, ultimately resulting in an enhanced degree of coalescence [43,44]. Upon exposure to identical freeze–thaw cycles, the emulsions stabilized by WPI-FA nanoparticles (at WPI/FA ratios of 3:1 and 2:1) exhibited markedly smaller droplet sizes than their counterparts, confirming that a modest level of FA incorporation effectively enhanced the cryo-resistance of the emulsion system. This protective effect was attributable to the FA-induced enhancement in both protein conformational flexibility and steric hindrance, which collectively facilitate the formation of a thickened interfacial adsorptive layer that mitigates ice crystal-mediated structural disruption. This observation was consistent with the work of Huang et al. (2023) [45], who reported that the soy protein hydrolysate–tannic acid complex reinforced the interfacial architecture, thereby inhibiting protein aggregation and improving freeze–thaw resilience.

### 2.9. The Salt Stability of the Og/W Emulsion Stabilized by WPI-FA Nanoparticle

**Figure 8:**
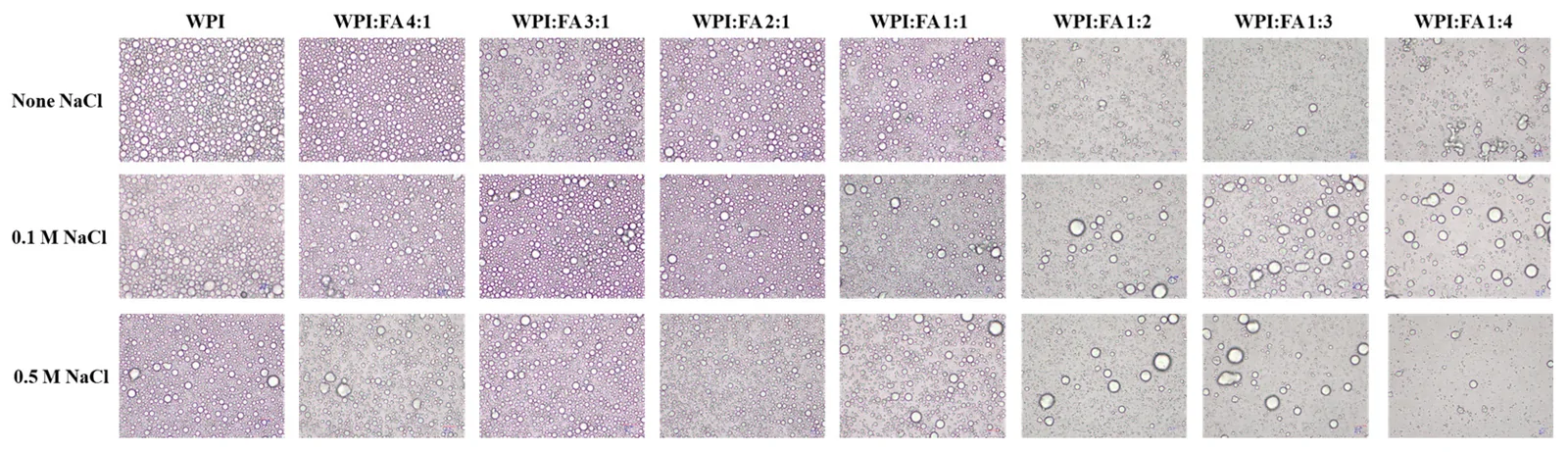
The salt stability of the emulsion stabilized by WPI-FA nanoparticles.

Given that salt is a common constituent of food systems, the stability of GLSO-based Og/W emulsion stabilized by WPI-FA nanoparticle was examined under different ionic strengths. Figure 8 shows the morphology of emulsions at ionic strengths ranging from 0 to 500 mM. For emulsions stabilized by WPI-FA nanoparticle with WPI/FA ratios of 3:1 and 2:1, the droplet size increased slowly as the salt ion concentration rose. When the FA proportion exceeded a 1:1 ratio (i.e., WPI/FA < 1:1), the droplet size increased significantly, with apparent flocculation and coalescence observed at high ionic strength. This phenomenon occurred because the reduced surface charge at higher salt concentrations weakened electrostatic repulsion between the protein coated GLSO droplets, making them more prone to aggregation via hydrophobic interactions among the interfacial proteins.

### 2.10. Rheological Characterization of Og/W Emulsion

**Figure 9:**
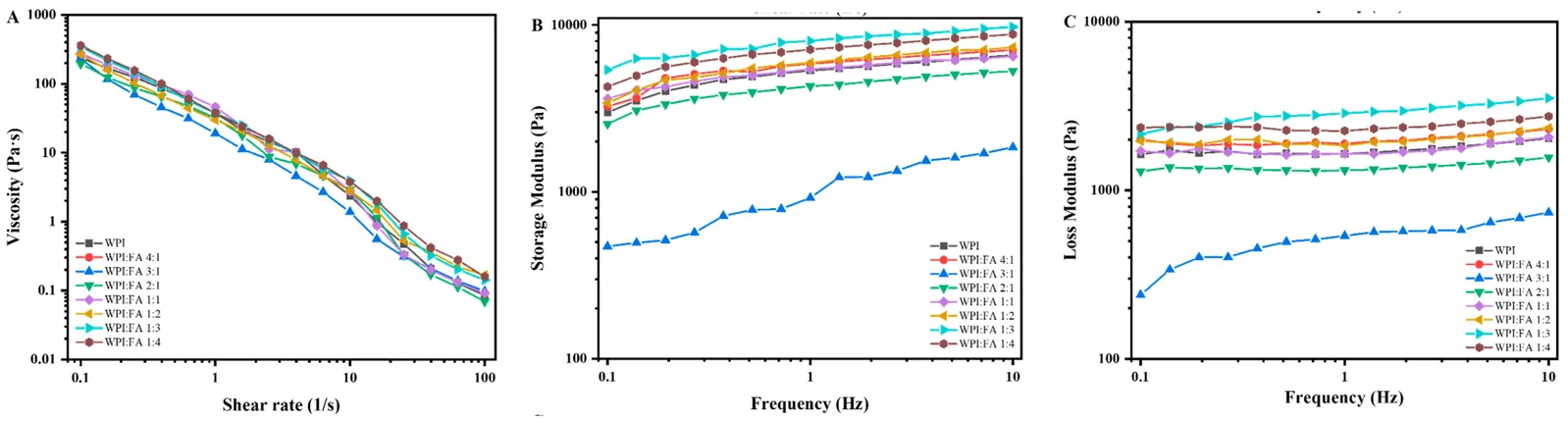
Apparent viscosity (**A**), storage modulus G′ (**B**) and loss modulus G″ (**C**) of the emulsions stabilized by WPI-FA nanoparticles.

Steady-shear viscosity measurements were performed on emulsions stabilized by WPI and WPI-FA nanoparticles with different WPI/FA ratios as a function of shear rate (Figure 9A). The shear rate dependence of viscosity fitted well the Carreau-Yasuda model (R^2^ > 0.99) (Table 1). All tested emulsions displayed typical shear-thinning characteristics, with viscosity declining as the shear rate increased. The flow behavior index (n), which quantifies the shear rate dependence of viscosity, was found to range from 0 to 1 for all samples, further verifying the pseudoplastic nature of these emulsions [46]. At a shear rate of 100 s^−1^, the viscosity of the emulsions followed the order: WPI/FA 1:4 ≈ WPI/FA 1:3 ≈ WPI/FA 1:2 > WPI/FA 1:1 ≈ WPI/FA 3:1 ≈ pure WPI ≈ WPI/FA 4:1 ≈ WPI/FA 2:1 (Figure 9A). Higher FA concentrations could induce bridging and depletion flocculation of droplets, which facilitated the formation of a denser network structure, strengthens inter-droplet interactions, and consequently increases the apparent viscosity of emulsions [47]. The storage modulus (G′) is an indicator of resistance to elastic deformation and a measure of the behavior of quasi-elastic solids. The loss modulus (G″) is the ratio of stress to strain under vibration conditions and is called the viscous response index. Frequency-sweep profiles showed that both G′ and G″ of all samples increased with frequency. G′ remained consistently higher than G″ across the entire frequency range tested, demonstrating a predominantly elastic response and the formation of an elastic-dominant, weak gel-like structure.

### 2.11. Intelligent Sensory Analysis of Emulsions

**Figure 10:**
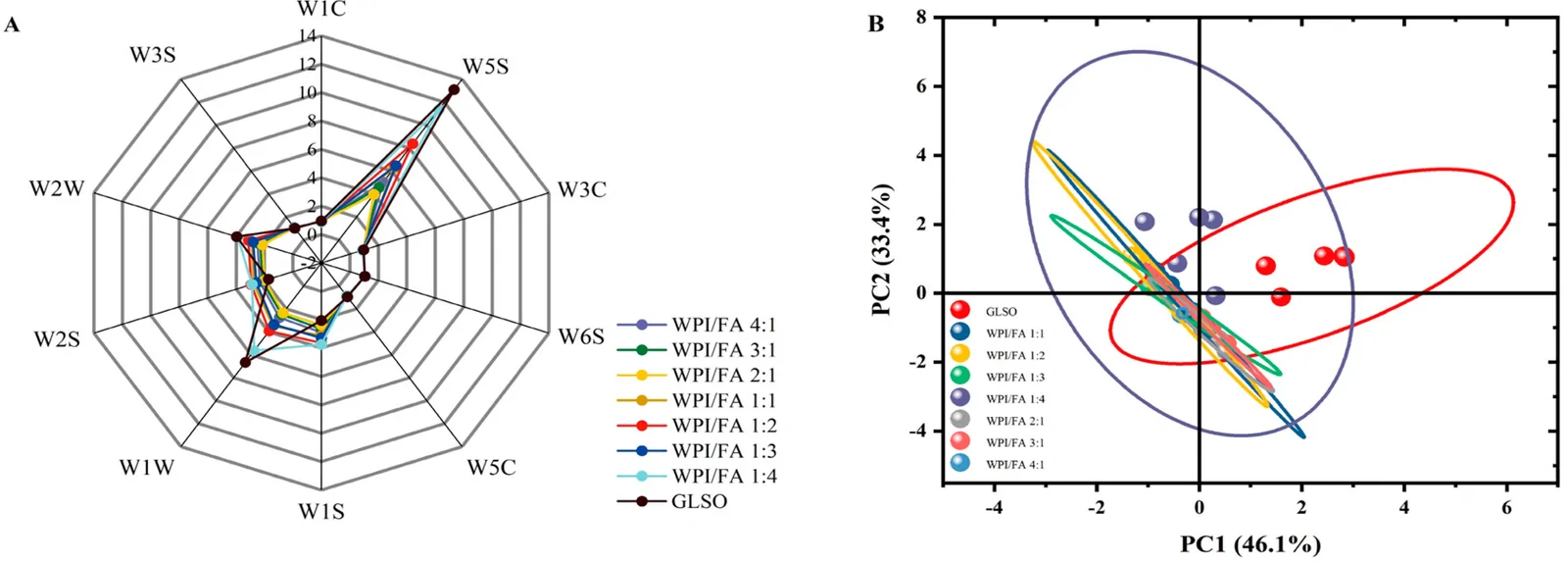
Relative intensity of each sensor of the electronic nose (**A**), principal component analysis of the electronic nose (**B**) with GLSO and emulsions stabilized by WPI-FA nanoparticles with different mass ratios of WPI to FA (4:1, 3:1, 2:1, 1:1, 1:2, 1:3, 1:4).

Since flavor and aroma characteristics heavily dictate consumer choices, identifying the specific aromatic traits of different emulsion variations is of profound practical merit. The volatile organic compounds of GLSO and its derived Og/W emulsion were profiled via an electronic nose array consisting of ten metal oxide sensors (W1C, W5S, W3C, W6S, W5C, W1S, W1W, W2S, W2W, W3S). According to the radar diagram (Figure 10A), GLSO exhibited a clear enrichment in aromatic hydrocarbons, sulfur-containing organic matter, and nitrogen oxides, as manifested by the prominent spikes in sensors W1W, W2W, and W5S. Previous research indicated that prolonged air exposure accelerates the oxidation of bioactive components in GLSO, such as terpenoids, polyphenols, and fatty acids. This oxidative degradation led to severe nutrient loss alongside the release of volatile compounds, including alkyl aromatics and nitrogen dioxide [48]. Compared with GLSO, the WPI-FA nanoparticle-stabilized Og/W emulsions exhibited markedly reduced signal profiles on sensors W1W, W2W, and W5S, suggesting a lower prevalence of sulfur-bearing organic compounds and nitrogen oxides. The results indicated that the WPI-FA nanoparticle emulsifier provided steric hindrance, thereby inhibiting the release of volatile odors associated with the oxidation of GLSO. These odor values tend to rise alongside increasing ferulic acid content. Notably, samples with WPI/FA ratios of 2:1 exhibited the least pronounced oily aroma. Given the identical GLSO concentration across all emulsion formulations, the observed discrepancy in headspace aroma intensity was primarily driven by the distinct stabilizer compositions. This is consistent with previous studies [49,50].

To evaluate these volatile profiles, principal component analysis (PCA) was executed. The first two principal components, PC1 (horizontal axis) and PC2 (vertical axis), accounted for 62.59% and 32.26% of the total variance, respectively, yielding a cumulative explanatory power exceeding 90% (Figure 10B). Consequently, this two-dimensional PCA framework successfully captured the predominant volatile attributes of the emulsion variations. These findings validated the feasibility of discriminating emulsions prepared with diverse stabilizers via electronic nose profiling. Collectively, the 2:1 WPI/FA ratio optimized the Og/W emulsion stability, thereby showing a inhibitory effect on GLSO volatilization.

## 3. Conclusions

In conclusion, this study systematically elucidated the molecular mechanisms by which FA modulated the structural attributes of WPI and enhanced the environmental stability of GLSO-based Og/W emulsions. Molecular dynamics simulations and FTIR spectroscopy revealed that FA interacts non-covalently with WPI subunits. Specifically, FA was associated with α-lactalbumin via hydrogen bonding and hydrophobic interactions. Meanwhile, it was embedded into the hydrophobic cavity of β-lactoglobulin through a “lock-and-key” mechanism. These interactions induced the protein to unfold from ordered α-helix and β-sheet structures into flexible random coils. Optimizing the WPI/FA mass ratio to 2:1 yielded nanoparticles that minimized emulsion droplet size and maximized interfacial coverage, conferring exceptional tolerance to freeze–thaw cycles, high salinity, and effectively suppressing the volatilization of GLSO flavors. Future research warrants a deeper investigation into how this protective WPI–FA interfacial layer and its antioxidant synergy encapsulate lipophilic bioactives, such as ganoderic acids. Specifically, the exact mechanisms preventing gastrointestinal degradation and ensuring targeted intestinal release should be systematically evaluated. Such studies will be crucial to fully validate this system for high-end nutraceuticals, functional beverages, and pharmaceutical controlled-release carriers.

## 4. Materials and Methods

### 4.1. Materials

*Ganoderma Lucidum* spores oil was purchased from Jinzhai Qiaokang Pharmaceutical Co., Ltd. (Lu’an, China). The analytical-grade 80% pure whey protein isolate (WPI) and ferulic acid (FA) were obtained from Yuanye Bio-Technology Co., Ltd. (Shanghai, China). Glyceryl Monostearate was obtained from Dibai Biotechnology Co., Ltd. (Shanghai, China). Ethyl alcohol, Sodium hydroxide, hydrochloric acid, sodium chloride was of analytical grade.

### 4.2. Preparation of WPI-FA Nanoparticles

Whey protein isolate (WPI) solution (1.0%, *w*/*v*) was prepared by dissolving WPI powder in Milli-Q water under continuous stirring for 2 h, followed by refrigeration at 4 °C for 24 h for complete hydration. Prior to use, the solution pH was adjusted to 7.0. The WPI solution and FA ethanol solution were then mixed at WPI-to-FA mass ratios ranging from 4:1 to 1:4 and stirred with a magnetic stirrer (SN-MS-6D, Shangpu Instrument Equipment, Shanghai, China) for 1 h. The resulting mixture was subjected to rotary evaporation (N-1300, Ailang Instrument, Shanghai, China) at 40 °C and 100–150 mbar to remove ethanol until no further solvent reflux was observed and constant weight was achieved, yielding a suspension of WPI-FA nanoparticles. The suspension was subsequently freeze-dried in a freeze dryer (FD-1A-80, Bilang Experimental Instrument, Tianjin, China) with an ice condenser temperature of −60 °C and vacuum level of 4 Pa for 24 h to obtain the WPI-FA nanoparticles in dry powder form. As a control, pure WPI particles were prepared following the same procedure described above, omitting the addition of FA.

### 4.3. Preparation of GLSO Oleogels in Water Emulsion

Lyophilized WPI–FA nanoparticles were re-dissolved in water to obtain a 3% (*w*/*v*) dispersion. Meanwhile, GLSO oleogels were prepared following the method of Zheng et al. (2020) [51]. Briefly, glyceryl monostearate (GM) was mixed into 10 mL of GLSO to obtain a 4% mixture, which was stirred in a 70 °C water bath for 20 min until transparent. Oleogel-in-water emulsions were then fabricated according to the method described by Shang et al. (2023) [29]. The WPI–FA nanoparticle dispersion and the preheated oleogels were homogenized using a T25 digital ULTRA-TURRAX (IKA, Staufen, Germany) at 7000 rpm for 2.5 min to yield emulsions with a 50% oleogel phase volume fraction. An emulsion stabilized solely by native WPI served as the control. For comparison, control emulsions were prepared under identical conditions using pure WPI particles as the emulsifier instead of WPI-FA complex particles. All other parameters, including oil-to-water ratio and homogenization conditions, remained unchanged.

### 4.4. Particle Size and ζ-Potential of WPI-FA Nanoparticles

The volume-weighted mean diameter d_4,3_ of the WPI-FA nanoparticles and the prepared emulsions was determined utilizing a Malvern Zetasizer Nano instrument (Ver 6.30, Malvern Instruments Ltd., Malvern, UK). In short, 1 mL of each specimen was transferred into a disposable cuvette and allowed to thermally equilibrate within the testing chamber at 25 °C for 5 min. The resulting particle size distribution data were subsequently used to extract the d_4,3_ values. The ζ-potential value is an average value measured five times to minimize data errors.

### 4.5. Contact Angle of Oil-Water Interface of WPI-FA Nanoparticles

To evaluate wetting behavior, the lyophilized sample was compressed into a thin pellet and secured inside a GLSO-phase cuvette. Subsequently, a 5 μL droplet of deionized water was deposited onto the pellet surface at a controlled temperature of 25 °C, with the resulting droplet profiles captured via an optical contact angle meter (Theta, Biolin Scientific, Gothenburg, Sweden) [52].

### 4.6. Turbidity of WPI-FA Nanoparticles

The turbidity of the prepared nanoparticles was reflected by monitoring its absorbance at a wavelength of 600 nm following a 50-fold dilution.

### 4.7. Fourier Transform Infrared of WPI-FA Nanoparticle

Fourier transform infrared (FTIR) measurements were carried out using a Nicolet 6700 spectrometer (Thermo Fisher Scientific, Waltham, MA, USA). In brief, sample pellets were prepared by mixing 1 mg of the material with 100 mg of KBr. Spectra were recorded over the range of 4000–400 cm^−1^, with 64 scans and a spectral resolution of 4 cm^−1^. Curve-fitting of the Amide I band (1600–1700 cm^−1^) was performed using PeakFit software (Systat PeakFit 4.12, SeaSolve Software Inc., San Jose, CA, USA). Prior to fitting, each spectrum was baseline-corrected and smoothed. Second-derivative spectroscopy was applied to identify the number and positions of the underlying component bands. The relative percentages of α-helix, β-sheet, β-turn, and random coil were then calculated from the integrated areas of the corresponding sub-peaks after Gaussian/Lorentzian curve-fitting.

### 4.8. Molecular Docking

Molecular docking was conducted via AutoDock to investigate the interactions of FA with α-lactalbumin (α-LA, PDB: 1F6S) and β-lactoglobulin (β-LG, PDB: 3BLG), the major components of whey protein isolate. The FA structure (CAS: 1135-24-6) was retrieved from PubChem. Receptors were prepared by removing crystallographic waters, adding hydrogens, and assigning Gasteiger charges in AutoDockTools 1.5.7. Docking simulations utilized the local search algorithm, and the lowest-energy binding poses were identified and visualized using PyMOL 4.6.0.

### 4.9. Molecular Dynamics Simulation

Molecular dynamics (MD) simulations were executed via GROMACS 2024.3 (Yan et al., 2026) [53], utilizing the top-ranked docking poses as starting configurations. The Amber 14SB force field and TIP3P model were applied to characterize the proteins and water molecules, respectively, while the ligand FA was parameterized using the General Amber Force Field (GAFF) with RESP2 charges. To neutralize the system, counterions (Na^+^ or Cl^−^) were introduced into the cubic, periodically boxed solvent environment. System minimization proceeded sequentially through steepest descent and conjugate gradient algorithms until the maximum force fell below 100 kJ/(mol·nm). Subsequently, a 200 ps NVT equilibration and a 1000 ps NPT equilibration were conducted. Throughout these phases, temperature (298.15 K) and pressure (1 bar) were regulated by the V-rescale thermostat and Parrinello–Rahman barostat, respectively. Finally, a 150 ns production MD run was performed using a 2-fs time step for a total of 7.5 × 10^7^ steps.

### 4.10. Microstructure Observation of WPI-FA Nanoparticles

Following the protocol of Yan et al. (2024) [54], freeze-dried samples were mounted on double-sided conductive tape and gold-sputtered for 60 s. The microstructures were then characterized using a scanning electron microscope (SU8010, Hitachi Inc., Tokyo, Japan) with a magnification of 30,000×.

### 4.11. Determination of Antioxidant Activity of WPI-FA Nanoparticles

The WPI-FA nanoparticle suspension was diluted 10-fold with distilled water for the determination of antioxidant properties. The DPPH radical scavenging activity, ABTS cation radical scavenging activity, and total antioxidant capacity (T-AOC) of the WPI-FA nanoparticle solution were measured [55,56].

#### 4.11.1. DPPH Free Radical Scavenging Activity Assay

For DPPH radical scavenging measurement, the blank (A_0_) contained 30 μL of 70% methanol and 70 μL of DPPH solution, incubated in the dark for 30 min. For samples, 30 μL of nanoparticle solution was mixed with 70 μL of DPPH solution (A_1_), while the background control used 30 μL of sample with 70 μL of 70% methanol (A_2_). Absorbance was read at 517 nm using a spectrophotometer, with each concentration tested in triplicate. Vitamin C served as the positive control under identical conditions. The scavenging rate was calculated as follows:

(1) $$DPPH\;scavenging\;effect\;(\%)=\;\frac{A_{0}-\left(A_{1}-A_{2}\right)}{A_{0}}\times 100$$

#### 4.11.2. ABTS Radical Scavenging Activity Assay

The ABTS radical cation decolorization assay was performed at 405 nm. In brief, the ABTS working solution was reacted with each sample aliquot under dark conditions. Three absorbance measurements were recorded: A0 (blank, ABTS solution plus solvent), A1 (sample reaction, ABTS solution plus sample), and A2 (sample background, sample plus solvent). All tests were carried out in triplicate, and ascorbic acid was included as a positive control. The radical scavenging activity was then calculated using the formula provided.

(2) $$ABTS\;radical\;scavenging\;rate\;\left(\%\right)=\left[1-\left(A_{1}-A_{2}\right)/A_{0}\right]\times 100$$

#### 4.11.3. Reducing Capacity Assay of the Quantified Constituents (T-AOC)

The antioxidant reducing power of nanoparticle solution was determined with a total antioxidant capacity assay kit (Solarbio, Beijing, China, BC1315) following the supplied protocol. The assay principle involves the acidic reduction in the Fe^3+^-TPTZ chromophore to the colored Fe^2+^-TPTZ complex, detected at 593 nm. This system enabled a side-by-side comparison of the ferric-reducing ability among individual reference compounds under uniform conditions. Results were calculated based on the final Fe^2+^ concentration produced in each reaction.

### 4.12. Salt Ion Stability of WPI-FA Nanoparticles

The resistance of the WPI-FA nanoparticle to ionic salts was examined. Briefly, a 1 mL aliquot of the nanoparticle solution (0.5%) was mixed with 1 mL of NaCl solutions of different concentrations (0, 0.1 M, 0.3 M, and 0.5 M). The samples were held at room temperature for 12 h, after which the volume-weighted mean diameter (d_4,3_) of the WPI-FA nanoparticles.

### 4.13. Freeze–Thaw Stability of Og/W Emulsion

Assessment of the freeze–thaw stability for the freshly made emulsion was conducted following a methodology optimized from Zhang et al. (2021) [57]. Briefly, 10 mL aliquots of the emulsion were transferred into screw-cap glass vials and sealed tightly to prevent solvent evaporation and external contamination during the temperature cycles. The freeze-thaw challenge consisted of 2 consecutive cycles, each comprising freezing at −20 °C for 24 h followed by thawing under ambient conditions for 4 h.

### 4.14. Rheological Measurement of Og/W Emulsion

The steady-shear flow behavior of the emulsions was characterized on a Discovery HR-2 rheometer (TA Instruments, New Castle, DE, USA), following the procedure outlined by Yan et al. (2022) [58]. All viscosity data were acquired with a 40 mm parallel plate fixture. Flow ramp tests were carried out at a constant temperature of 25 °C, with shear rates ranging from 0.01 to 100 s^−1^. The resulting flow curves were then modeled using the Carreau Yasuda equation, expressed as:

(3) $$\eta =\eta_{\infty }+\frac{\eta_{0}\;-\;\eta_{\infty }}{{\left(1\;+\;{\left(\frac{\gamma ˙}{{\gamma ˙}_{\;c}}\right)}^{p}\right)}^{1-n}}$$

Here, η is the apparent viscosity (Pa·s), η_0_ the zero shear viscosity (Pa·s), η∞ the infinite shear viscosity (Pa·s), *γ*˙ the shear rate (s^−1^), and n the dimensionless power law index. *γ*˙*c* represents the critical shear rate. *p* is a dimensionless exponent. Separately, the emulsions’ dynamic viscoelastic behavior was probed at 25 °C via frequency sweeps from 0.1 to 10 Hz at a constant 0.1% strain, which was verified to be inside the linear viscoelastic region.

### 4.15. Salt Ion Stability of Og/W Emulsion

The resistance of the freshly made emulsion to ionic salts was examined. Briefly, a 1 mL aliquot of the emulsion was mixed with 1 mL of NaCl solutions of different concentrations (0, 0.1 M, and 0.5 M). The samples were held at room temperature for 12 h, after which the emulsion morphologies were visualized.

### 4.16. E-Nose Analysis of Samples of Og/W Emulsion

Volatile flavor profiles of the samples were characterized utilizing a PEN3 electronic nose system (Airsense Analytics, Schwerin, Germany), which integrates 10 distinct metal oxide semiconductor sensors. The specific selectivity of these sensors includes nitrogen oxides (W5S), sulfides and organic sulfur compounds (W1W), alongside organic sulfides and aromatic molecules (W2W). The remaining sensors (W1C, W3C, W6S, W5C, W1S, and W3S) primarily respond to alkanes, alcohols, and aromatic compounds. For the headspace analysis, 0.3 g of each sample was sealed in a 20 mL vial and allowed to equilibrate at ambient temperature for 30 min. After a 180 s system purging process, sampling was conducted via dual probes (one linked to the sensor chamber and the other to a carbon filter). The response data gathered from 100 to 110 s were extracted for subsequent statistical evaluation.

### 4.17. Statistical Analysis

All experimental measurements were performed in triplicate, and the resulting data are presented as means with their corresponding standard deviations. Statistical comparisons among groups were conducted using SPSS 21.0 (SPSS Institute, Cary, NC, USA), with significance set at a *p* level of 0.05, and mean separations were assessed by Duncan’s multiple range test. Principal Component Analysis (PCA) was performed using OriginPro 2021 with autoscaling pre-processing, and principal components were extracted based on Kaiser’s criterion.

## Figures and Tables

**Table 1:** Carreau model equation fitting parameters of the emulsion stabilized by WPI and WPI-FA nanoparticles.

Samples	η_0_ (Pa·s)	η_∞_ (Pa·s)	γ˙c (s^−1^)	n	R^2^
WPI	210.19 ± 1.93	0 ± 0.98	0.158 ± 0.011	0.07 ± 0.05	0.999
WPI/FA 4:1	216.35 ± 4.53	0 ± 2.94	0.151 ± 0.014	0.14 ± 0.08	0.996
WPI/FA 3:1	214.09 ± 4.07	0 ± 1.78	0.070 ± 0.006	0.14 ± 0.07	0.995
WPI/FA 2:1	185.91 ± 2.87	0 ± 1.45	0.134 ± 0.029	0.10 ± 0.12	0.998
WPI/FA 1:1	220.31 ± 4.49	0 ± 2.28	0.150 ± 0.015	0.13 ± 0.08	0.996
WPI/FA 1:2	228.79 ± 2.09	0 ± 0.99	0.104 ± 0.005	0.12 ± 0.04	0.999
WPI/FA 1:3	307.99 ± 0.91	0 ± 0.41	0.117 ± 0.002	0.16 ± 0.04	0.999
WPI/FA 1:4	324.00 ± 1.94	0 ± 0.88	0.125 ± 0.005	0.06 ± 0.03	0.999

## Data Availability

The original contributions presented in this study are included in the article. Further inquiries can be directed to the corresponding author.
